# Microanalysis and Optimization of Sulfate Corrosion Resistance of Flowable Concrete Using Response Surface Methodology

**DOI:** 10.3390/ma19183963

**Published:** 2026-09-18

**Authors:** Wenhua Yuan, Min Fang, Haoran Zhai, Jiaying Yang

**Affiliations:** 1 State Key Laboratory for Safe Mining of Deep Coal Resources and Environmental Protection, Huainan 232000, China; 2 School of Civil Engineering and Architecture, Anhui University of Science and Technology, Huainan 232001, China; 19956841079@163.com (H.Z.);; 3 China Energy Engineering Group Anhui No.2 Electric Power Construction Co., Ltd., Hefei 230601, China; 13855969540@163.com

**Keywords:** flowable concrete, response surface methodology, workability, compressive strength, sulfate corrosion resistance, microanalysis

## Abstract

In this study, the Box–Behnken response surface method was used to investigate the effects of coarse aggregate gradation (percentage of 5–10 mm stone), water-reducing agent mixture, and aggregate-to-cementitious material ratio on the workability, compressive strength, and sulfate corrosion resistance of flowable concrete. These three factors served as the independent variables. A response surface regression model was constructed using data from 17 tests to analyze the variations in slump flow, J-ring extensibility, 28-day compressive strengths, compressive strength retention ratio under sulfate attack, and relative mass loss ratio. The results showed that coarse aggregate gradation (A) had the most significant effect on each performance index, followed by the aggregate-to-cementitious material ratio (C); the water-reducing agent mixture (B) had the least impact. The optimized proportions were determined as follows: 71.149% for the 5–10 mm stones, 1.695% for the water-reducing agent mixture, and an aggregate-to-cementitious material ratio of 2.749. Microanalysis revealed that during the initial stage of sulfate corrosion, ettringite (AFt) and gypsum were generated to fill the pore spaces and improve densification, resulting in an increase in quality and strength; however, prolonged corrosion led to expansion damage and accelerated deterioration of the concrete. Scanning electron microscope (SEM) tests further verified the correlation between the macroscopic and microstructure properties.

## 1. Introduction

Flowable concrete [1,2] offers construction efficiency through self-compaction, yet its service performance is critically governed by the interplay of mix-design parameters. Coarse aggregates account for approximately 30% of the total volume of flowable concrete, and their morphology, particle size, and grading determine not only the flow, filling, and stability of the fresh mix but also the mechanical and durability properties after hardening [3]. Wu et al. [4] studied the influence of coarse aggregate gradation on fair-faced self-compacting concrete based on the average mortar thickness theory, and found that optimal performance was achieved when the volume fraction of coarse aggregate was 35% and the gradation ratio (10–20 mm:5–10 mm) was 7:3.

Achieving both high fluidity and strength in concrete is challenging. When the cementitious material content is fixed, increasing the water–cement ratio improves fluidity but reduces strength. The use of high-efficiency water-reducing admixtures can resolve this conflict [5,6,7]. Shi et al. [8] investigated the effect of water-reducing agent on the workability of C40 flowable concrete using manufactured sand and found optimum fluidity and cohesion at a 1.1% dosage. Basu et al. [9] demonstrated that self-compacting concrete attained maximum strength when the water-reducing agent accounted for 1.35–1.7% of the cementitious material at a water–cement ratio of 0.34. Durability [10,11] is a critical property of concrete, denoting its capacity to maintain serviceability under extended environmental exposures.

Among the four factors that most significantly influence concrete durability—sulfate attack, alkali–aggregate reaction, reinforcement corrosion, and freeze–thaw cycles [12]—sulfate attack is particularly harmful, especially in salinized areas, regions with heavy industrial wastewater discharge, and coastal zones. Salinized land encompasses approximately 950 million hectares, constituting about 7.3% of the planet’s total terrestrial area, with China ranking as the third most affected nation [13]. Salinized soil contains high concentrations of soluble salts, among which sulfate ions are particularly aggressive to concrete, causing chemical deterioration through the formation of expansive products such as ettringite and gypsum. Consequently, concrete structures in such environments are vulnerable to significant damage from sulfate attack [14].

Durability [10,11] is a critical property of concrete, denoting its capacity to maintain satisfactory serviceability and an intact appearance when subjected to environmental exposures over extended periods. Concrete durability is a critical factor in determining structural integrity and safety. Poor durability can lead to premature aging and structural damage, thereby compromising the integrity of buildings and threatening the safety of people and property. Consequently, considerable attention has been given to the long-term durability of concrete structures. The four factors that most significantly influence concrete durability are sulfate corrosion, alkali–aggregate reaction, reinforcement corrosion, and freeze–thaw cycles [12]. Among these, sulfate corrosion is particularly harmful, especially in areas affected by salinization, substantial industrial wastewater discharge, and coastal regions. The damage caused by sulfate corrosion is especially significant in these areas. Examining the phenomenon of salinized land [13] reveals that such land encompasses approximately 950 million hectares, constituting approximately 7.3% of the planet’s total terrestrial area. China ranks as the third most affected nation, with an estimated 100 million hectares of salinized land spread over 19 provinces, including the northeastern, northwestern, and coastal regions. Salinized soil contains high concentrations of soluble salts, including sulfates and chlorides. Consequently, concrete structures on such land are subjected to significant damage from sulfate attack [14]. In view of these points, it is imperative to develop concrete with robust resistance to sulfate corrosion.

The Response Surface Methodology (RSM), first proposed by Box and Wilson in 1951 [15], is an experimental design method that constructs a mathematical model between independent variables and response values to predict response changes and determine optimal solutions [16,17]. RSM offers advantages, including reduced trials, enhanced accuracy, lower cost, and the ability to analyze complex factor interactions through intuitive three-dimensional response surface and contour plots [18,19], and has been increasingly applied in concrete research [20,21]. For instance, Ujwal et al. [22] employed RSM to optimize self-compacting concrete incorporating fish-scale powder, examining the effects of powder dosage and water–cement ratio on workability and strength. Mahmoud et al. [23] used central composite RSM to optimize SCC mixes with limestone powder, PET, and superplasticizer, focusing on fresh and mechanical properties. Safari et al. [24] applied RSM to SCC containing rice husk ash and fibers, investigating workability and compressive strength development. However, most existing RSM studies on concrete have focused on single-performance indicators or isolated factors, with limited attention to simultaneous optimization involving durability, particularly sulfate corrosion resistance, under the combined effects of coarse aggregate gradation, water-reducing agent dosage, and aggregate-to-cementitious material ratio. This study therefore employs the Box–Behnken response surface design to systematically investigate these three factors and five response variables, aiming to fill this research gap.

Current research on factors affecting the sulfate corrosion resistance of concrete mainly focuses on sulfate concentration, water-reducing agent mixtures, and admixtures; however, investigations into coarse aggregate gradation, water-reducing agent mixtures, and the aggregate-to-cementitious material ratio are limited. Many studies have examined sulfate corrosion resistance solely by deriving an optimal mix ratio, without incorporating it into an overall mix optimization framework. Therefore, in this study, the Box–Behnken response surface test was designed for the sulfate corrosion resistance of flowable concrete to construct a response surface model for workability, compressive strength, and sulfate corrosion resistance; to study the effects of coarse aggregate gradation, water-reducing agent mixture, and aggregate-to-cementitious material ratio on flowable concrete; and to derive the optimal mix ratio. This study provides theoretical support and an optimization basis for the durability design of concrete in salinized land, areas with severe industrial wastewater discharge, and coastal regions.

## 2. Materials and Methods

### 2.1. Materials

The cement selected was P·II52.5 Portland cement, conforming to GB 175-2023, supplied by China Conch Cement Co., Ltd. (Wuhu, China), with a density of 3.15 g/cm^3^ and a specific surface area of 368 m^2^/kg. Mineral admixtures were Grade I fly ash and microsilica, with chemical compositions as follows: silica fume was mainly composed of SiO_2_ 96.3%, and fly ash contained SiO_2_ 43% and Al_2_O_3_ 24%. Coarse aggregates consisted of 5–10 mm and 10–20 mm gravel, with apparent densities of 2680 kg/m^3^ and 2740 kg/m^3^, respectively; the fine aggregate was sand with a fineness modulus of 2.64 and a mud content of 0.26%. The water-reducing agent was a polycarboxylic acid high-performance water-reducing agent with a solid content of 39% and a water-reducing rate of 28%; it is a polycarboxylate ether-based superplasticizer produced by Sobute New Materials Co., Ltd. (Nanjing, China). The water used was domestic tap water.

### 2.2. Methods

#### 2.2.1. Macroscopic Test Methods

Concrete workability tests, including the slump flow test and the J-ring extensibility test, were conducted in accordance with the relevant methods specified in the JGJ/T 283-2012 Technical Specification for the Application of Flowable Concrete [25].

The compressive strength test was carried out according to the GB50081-2019 Standard for Test Methods of Concrete Physical and Mechanical Properties [26] using cubic specimens with a side of 100 mm. Three specimens were tested for each mixture at each age, and the average value was reported. The specimens were loaded at a rate of 0.8 MPa/s. The compressive strength was calculated using Equation (1) and then multiplied by a size conversion factor of 0.95 to convert the results from 100 mm cubes to the equivalent 150 mm standard cube strength. The results are reported accurate to 0.1 MPa.

(1) $$\begin{array}{c} F_{\mathrm{cc}}=F/A \end{array}$$

*F*_cc_—compressive strength of concrete cubes (MPa);

F—specimen destructive load (N);

A—pressurized area of the test block (mm^2^).

The sulfate corrosion resistance test was conducted per the GB/T50082-2024 Standard for Test Methods of Long-Term Performance and Durability of Ordinary Concrete [27]. Specimens were demolded 24 h after casting and immediately transferred to a standard curing room for 28 days of curing. After curing, the specimens were transferred to a 5% Na_2_SO_4_ solution at 20 ± 2 °C for further exposure. Three specimens were tested for each group at each age, and the average value was reported. The Na_2_SO_4_ solution was not renewed during the entire test period. The compressive strength retention ratio under sulfate attack and the mass loss ratio were chosen as evaluation indices for sulfate corrosion resistance. Their calculation formulas are given in Equations (2) and (3), respectively.

(2) $$K_{f}\;=\;f_{\mathrm{cu},n}/f_{\mathrm{cu},0}\;\times \;100\%$$

K_f_—compressive strength retention ratio under sulfate attack (%);

f_cu,n_—compressive strength of the specimen after immersion in a 5% Na_2_SO_4_ solution for n days (accurate to 0.1 MPa);

f_cu,0_—compressive strength of clear-water group test blocks of the same age (accurate to 0.1 MPa).

(3) $$\begin{array}{c} ∆w={(M}_{0}-M_{n})/M_{0}\times 100\% \end{array}$$

∆w—relative mass loss ratio (%);

M_0_—mass of clear-water group blocks of the same age, measured to the nearest 0.1 kg;

M_n_—mass of the test block after n days of immersion in a 5% Na_2_SO_4_ solution (accurate to 0.1 kg).

#### 2.2.2. Microscopic Test Methods

Microscopic tests were conducted to investigate the microstructural changes and reaction products of flowable concrete subjected to sulfate corrosion. Scanning electron microscopy (SEM) (ZEISS, Jena, Germany) was used to observe the morphology and distribution of hydration products and corrosion products, while X-ray diffraction (XRD) was used to identify the crystalline phases formed during sulfate exposure. The formation of gypsum and calcium aluminate (AFt) in the sulfate-immersed specimens could be identified by XRD, while SEM could be used to observe the associated changes in the concrete microstructure, the formation of crystals, changes in the C-S-H gel, and the development of microcracks. These microscopic observations were used to explain the changes in the macroscopic compressive strength retention ratio under sulfate attack and relative mass loss ratio.

The same drying procedure was applied to both sulfate-immersed and control specimens, ensuring that the observed differences between groups are attributable to sulfate exposure.

(1) Scanning electron microscope test

After the compressive strength test, approximately 1 cm^3^ of concrete was selected from the crushed test block. The selected sample was immersed in an alcohol solution for 24 h to terminate the hydration process. It was then placed in an oven at 60 °C for 6 h to remove moisture. After drying, the sample surface was subjected to gold sputter coating to improve its electrical conductivity and obtain a stable signal during SEM observation. The prepared specimen was then fixed onto the sample stage under vacuum conditions and examined by SEM. The SEM images were used to observe the morphology, distribution, and interrelationship of the hydration and sulfate-erosion products in the concrete matrix.

(2) X-ray diffraction test

After the compressive strength test, representative concrete fragments were collected and immersed in an alcohol solution for 24 h to terminate hydration. The samples were subsequently dried in an oven at 60 °C for 6 h. After drying, the concrete fragments were ground into a fine powder for XRD analysis. XRD patterns were recorded using a Bruker D8 Advance diffractometer (Bruker AXS, Karlsruhe, Germany) with Cu Kα radiation λ = 1.5406 Å, under standard operating conditions, with a scanning range of 5° to 90° and a scanning rate of 10°/min. A specimen containing 10% ZnO was prepared as the internal standard. The obtained diffraction patterns were used to identify the crystalline phases present in the concrete specimens and to compare the phase compositions of the sulfate-immersed and clear-water groups. In particular, changes in the diffraction peaks of calcium hydroxide, gypsum, and AFt were used to evaluate the chemical reactions associated with sulfate corrosion.

### 2.3. Mixing Ratio Design

Based on the optimal ranges derived from a previous single-factor test [28], the proportion of 5–10 mm stones, water-reducing agent dosage, and aggregate-to-cementitious material ratio were used as the influencing variables. The center point of each factor was set at the empirically optimal value: 70% for 5–10 mm stone proportion, 1.7% for the water-reducing agent dosage, and 2.9 for the aggregate-to-cementitious material ratio. The ranges (60–80%, 1.5–1.9%, and 2.6–3.2) were selected to cover the practical operating window while ensuring all mixtures remained within acceptable workability and strength thresholds, and to provide sufficient spread for the RSM to accurately capture curvature and interaction effects. The response values included slump flow, J-ring extensibility, 28-day compressive strength, compressive strength retention ratio under sulfate attack, and mass loss ratio. The design of the Box–Behnken test comprised 17 groups—12 analytical factorization tests and 5 replicated tests. The test factors and levels are listed in Table 1, and the test mix ratios are listed in Table 2.

## 3. Results and Discussion

Table 3 lists the workability and compressive strength test results for flowable concrete. Values for the five replicated center-point trials are reported as mean ± standard deviation: slump flow = 668 ± 14.8 mm; J-ring extensibility = 643 ± 10.0 mm; 28-day compressive strength = 65.2 ± 1.6 MPa.

### 3.1. Response Surface Result Analysis

#### 3.1.1. Response Surface Modeling and Significance Analysis

Response surface regression model equations relating the coarse aggregate gradation, water-reducing agent mixture, and cement ratio to slump flow, J-ring extensibility, 28-day compressive strengths (Table 3), the compressive strength retention ratio under sulfate attack for the 90-day group (Table 4), and the relative mass loss ratio for the 90-day group (Table 5) were obtained using Design-Expert (Version 12) software, as shown in Equations (4)–(8). An ANOVA was used to test the significance of the model, and the results are presented in Table 6.

(4) Y_1_ = 668 − 12.5A + 6.25B − 5C + 1.25AB + 3.75AC − 6.25BC − 30.88A^2^ − 23.38B^2^ − 3.37C^2^

(5) Y_2_ = 643 − 16.25A + 2.5B − 6.25C − 2.5AB + 2.5AC − 7.5BC − 32.75A^2^ − 22.75B^2^ − 5.25C^2^

(6) Y_3_ = 65.2 + 3.89A + 1.18B − 2.91C − 0.85AB + 1.78AC + 4.15BC − 2.54A^2^ − 3.21B^2^ − 1.64C^2^

(7) Y_4_ = 87.32 + 1.09A + 0.2625B − 0.925C − 0.575AB + 0.15AC + 1.65BC − 1.5A^2^ − 0.7975B^2^ − 0.4225C^2^

(8) Y_5_ = 0.68 − 0.0625A − 0.0225B + 0.0275C − 0.02AB − 0.01AC − 0.02BC − 0.0275A^2^ − 0.0075B^2^ + 0.0825C^2^

As shown in Table 6, the model *p*-values are all lower than 0.05, the lack-of-fit *p*-values are all greater than 0.05, the R^2^ values are all above 0.9, and the difference between R_a_^2^ and R_p_^2^ is lower than 0.2, indicating that the model is highly credible [29,30,31].

#### 3.1.2. Response Surface Plot Analysis

(1) Slump flow analysis

**Figure 1:**
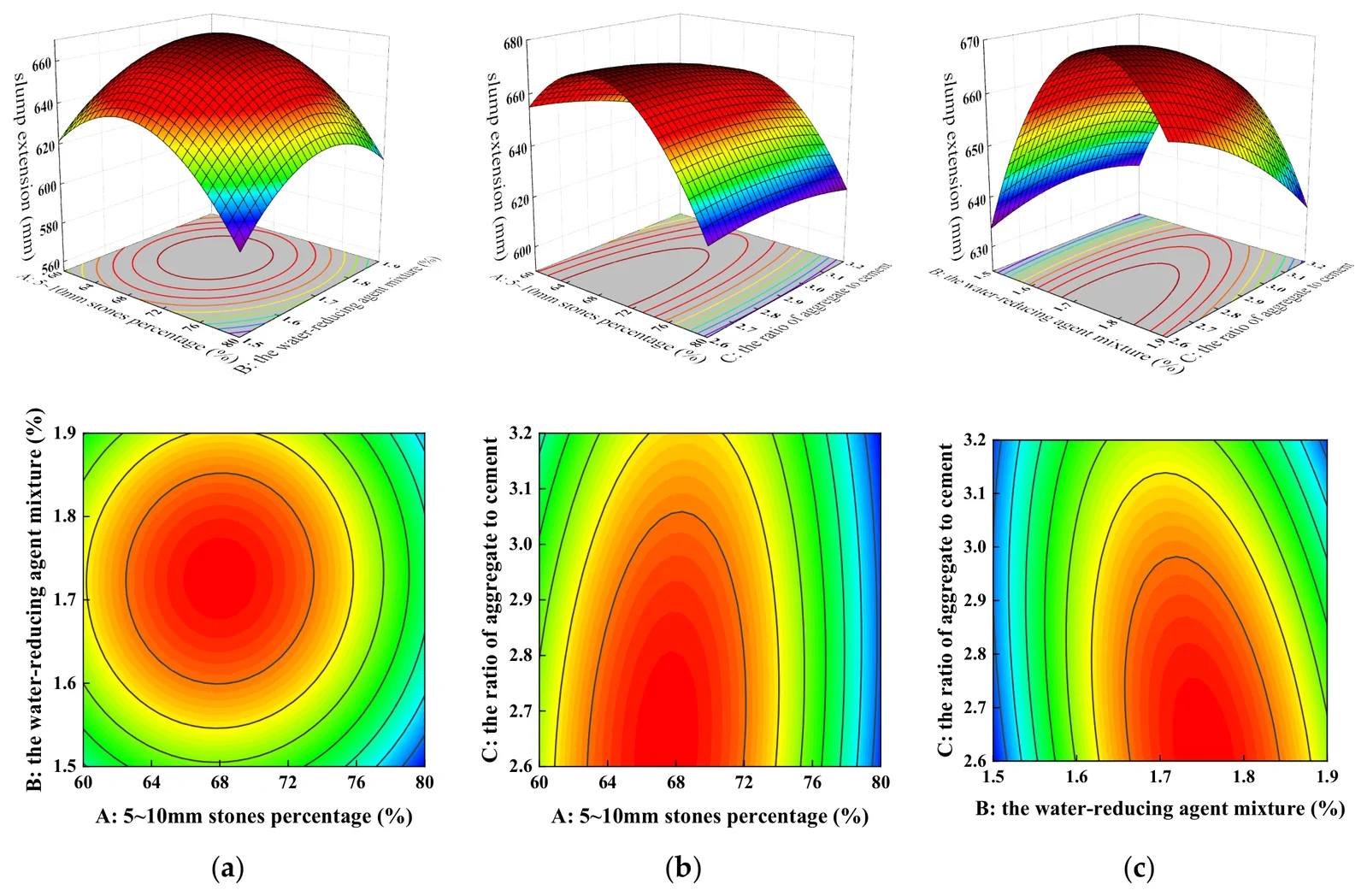
3D response surfaces and contour plots of slump flow: (**a**) A–B interaction, (**b**) A–C interaction, and (**c**) B–C interaction.

Figure 1 shows the three-dimensional (3D) response surface plots of slump flow and the corresponding contour plots. In Figure 1a, slump flow increases and then decreases with an increase in both the percentage of 5–10 mm stones and the water-reducing agent mixture. The curve for the 5–10 mm stone percentage exhibits a greater degree of curvature than that for the water-reducing agent mixture. In addition, the contour lines on the side representing the 5–10 mm stone percentage are denser, indicating that coarse aggregate gradation has a greater influence on slump flow. In Figure 1b, slump flow again increases and then decreases with an increase in the percentage of 5–10 mm stones; however, the influence of the aggregate-to-cementitious material ratio on slump flow is smaller. The denser contour lines on the side representing the 5–10 mm stone percentage further confirm that coarse aggregate gradation exerts a stronger influence on slump flow relative to the aggregate-to-cementitious material ratio. In Figure 1c, an increase in the water-reducing agent mixture initially increases slump flow and then decreases it, whereas an increase in the aggregate-to-cementitious material ratio causes a gradual decrease in slump flow. The contour lines are more densely distributed along the water-reducing agent mixture axis, suggesting that its effect on slump flow is greater than that of the aggregate-to-cementitious material ratio.

In summary, the degree of influence on slump flow of flowable concrete is ranked as A > C > B, indicating that increasing both the proportion of 5–10 mm stones and the aggregate-to-cementitious material ratio can effectively enhance slump flow. The ANOVA *p*-value verifies this conclusion. According to the ANOVA results in Table 6, the BC interaction term shows the lowest *p*-value among the three interaction terms for slump flow, followed by AC and AB. The response surfaces in Figure 1 illustrate these modeled trends.

(2) Analysis of J-ring extensibility

**Figure 2:**
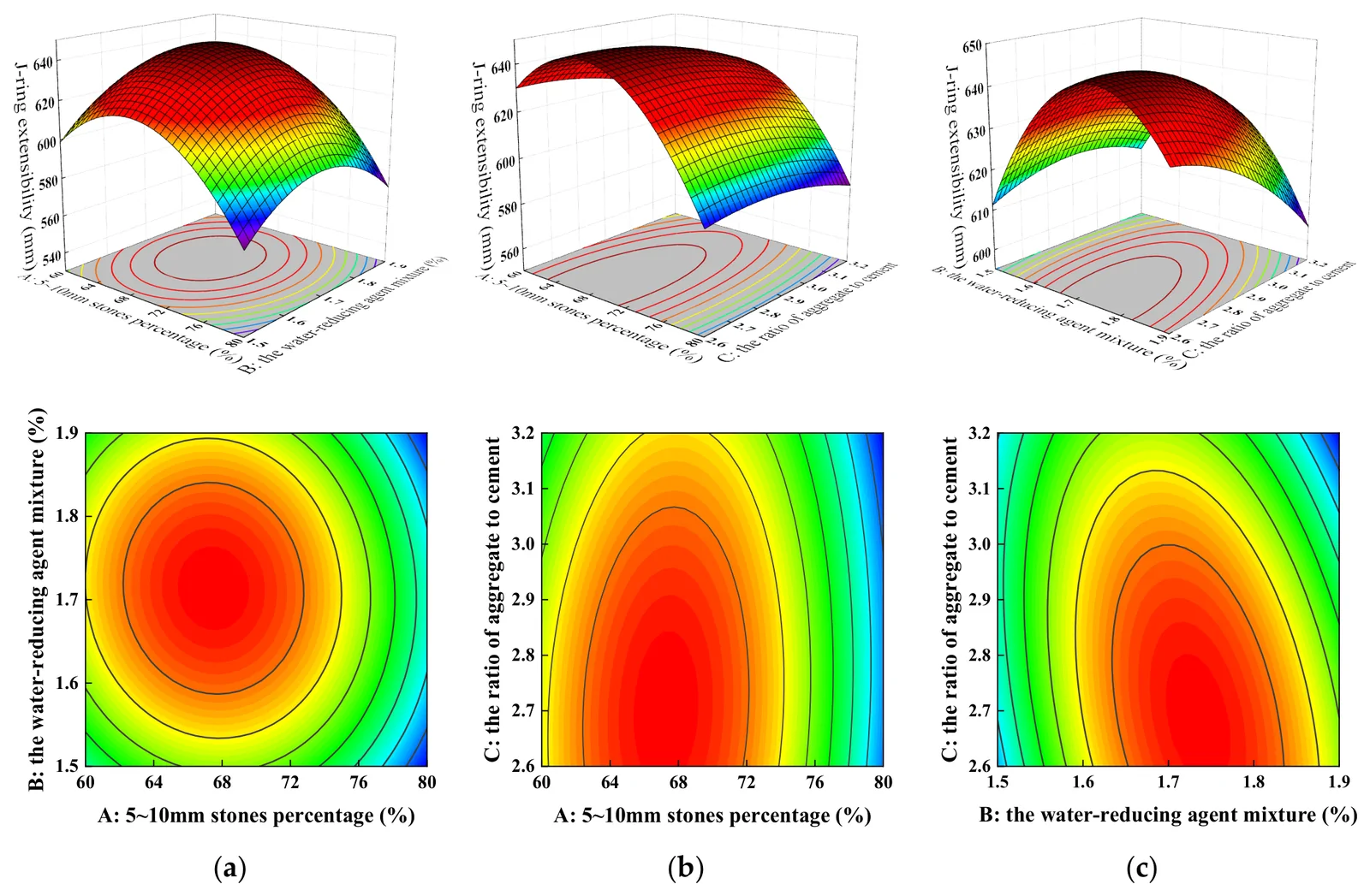
3D response surfaces and contour plots of J-ring extensibility: (**a**) A–B interaction, (**b**) A–C interaction, and (**c**) B–C interaction.

Figure 2 shows the 3D response surface and contour maps of J-ring extensibility. Figure 2a indicates that J-ring extensibility increases and then decreases with an increase in both the percentage of 5–10 mm stones and the water-reducing agent mixture. The curves for the 5–10 mm stone percentage are more curved, and the contour lines on the coarse aggregate gradation side are denser, indicating that the effect of the 5–10 mm stone percentage on J-ring extensibility is greater than that of the water-reducing agent mixture.

From Figure 2b, the response surface exhibits a downward-opening paraboloid, and J-ring extensibility initially increases and then decreases as the aggregate-to-cementitious material ratio increases; however, this trend is less pronounced compared to that observed for coarse aggregate gradation. The larger curvature for the coarse aggregate gradation and the denser contour lines confirm that its influence on J-ring extensibility is greater than that of the aggregate-to-cementitious material ratio.

Figure 2c presents a downward-opening paraboloid, where J-ring extensibility increases and then decreases with an increase in the water-reducing agent mixture, while it gradually decreases with an increase in the aggregate-to-cementitious material ratio. The curvature for the water-reducing agent mixture is larger than that for the aggregate-to-cementitious material ratio, and the contour lines along the aggregate-to-cementitious material ratio axis are denser than those along the water-reducing agent mixture axis, indicating that the aggregate-to-cementitious material ratio exerts a greater effect on J-ring extensibility than the water-reducing agent mixture.

In summary, the degree of influence on J-ring extensibility of flowable concrete is ranked as A > C > B, meaning that increasing both the proportion of 5–10 mm stones and the aggregate-to-cementitious material ratio can effectively improve J-ring extensibility and interstitial passing ability. According to the ANOVA results in Table 6, the BC interaction term shows the lowest *p*-value among the three interaction terms for J-ring extensibility. The response surfaces in Figure 2 illustrate the modeled interaction trends.

(3) 28-day compressive strength analysis

**Figure 3:**
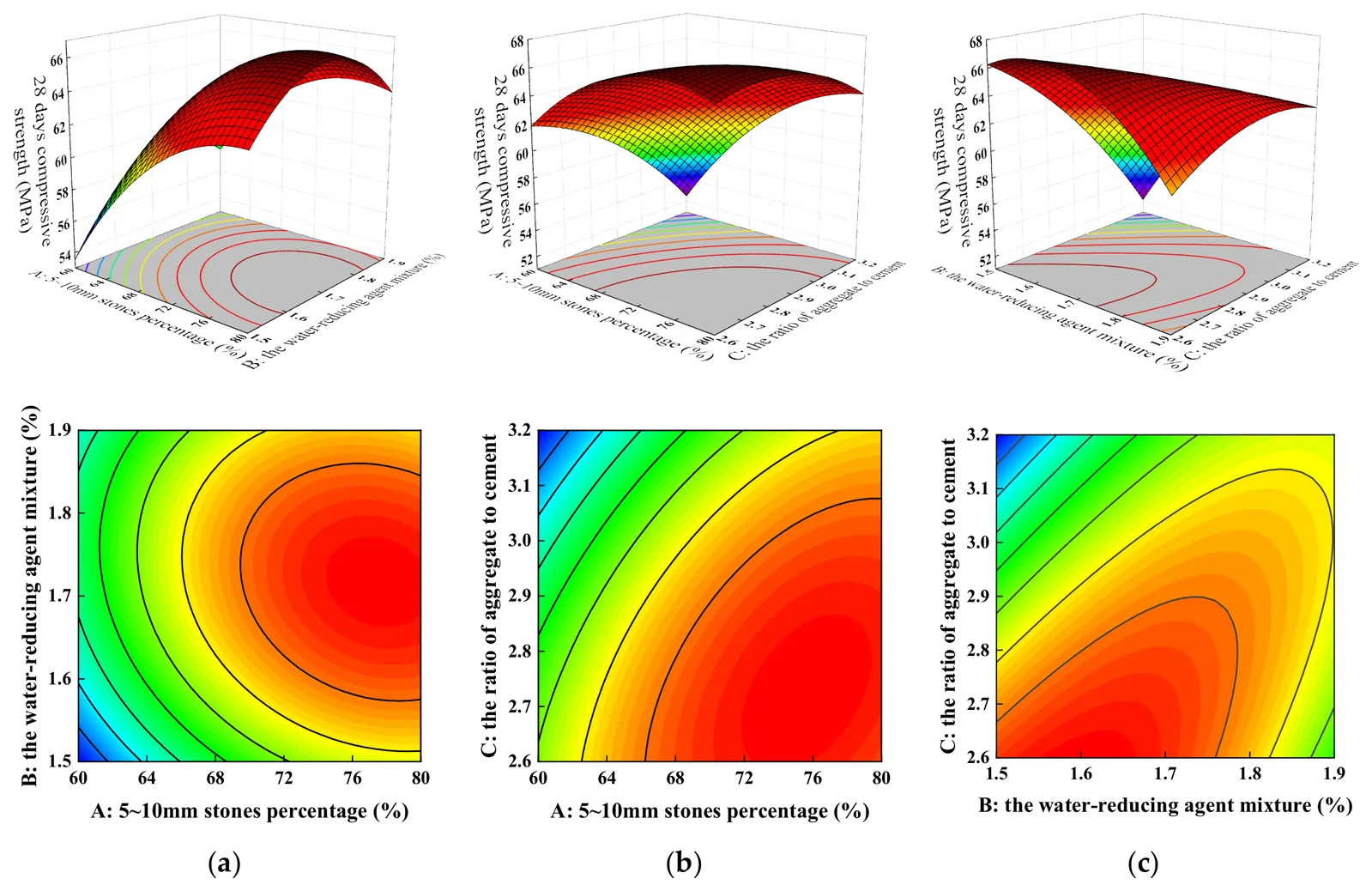
3D response surfaces and contour plots of 28-day compressive strength: (**a**) A–B interaction, (**b**) A–C interaction, and (**c**) B–C interaction.

Figure 3 shows the 3D response surface and contour plots of 28-day compressive strength. In Figure 3a, 28-day compressive strength exhibits an increasing then decreasing trend with an increase in the water-reducing agent mixture, while it continuously increases with an increase in the percentage of 5–10 mm stones. The denser contour lines on the coarse aggregate gradation side indicate that its effect on 28-day compressive strength is greater than that of the water-reducing agent mixture.

Figure 3b indicates that an increase in the percentage of 5–10 mm stones increases 28-day compressive strength, whereas an increase in the aggregate-to-cementitious material ratio decreases it. The denser contour lines on the coarse aggregate gradation side further demonstrate that its effect is more significant than that of the aggregate-to-cementitious material ratio.

Figure 3c shows that 28-day compressive strength increases and then decreases with an increase in the water-reducing agent mixture, while it decreases with an increase in the aggregate-to-cementitious material ratio. The contour lines along the aggregate-to-cementitious material ratio axis are denser, indicating that its influence on 28-day compressive strength is greater than that of the water-reducing agent mixture.

In summary, the influence on 28-day compressive strength of flowable concrete is ranked as A > C > B, meaning that increasing both the proportion of 5–10 mm stones and the aggregate-to-cementitious material ratio can effectively improve 28-day compressive strength. According to the ANOVA results in Table 6, the BC interaction term shows the lowest *p*-value among the three interaction terms for 28-day compressive strength, indicating that it is statistically significant. The response surfaces in Figure 3 illustrate these modeled trends.

(4) Compressive strength retention ratio under sulfate attack analysis

**Figure 4:**
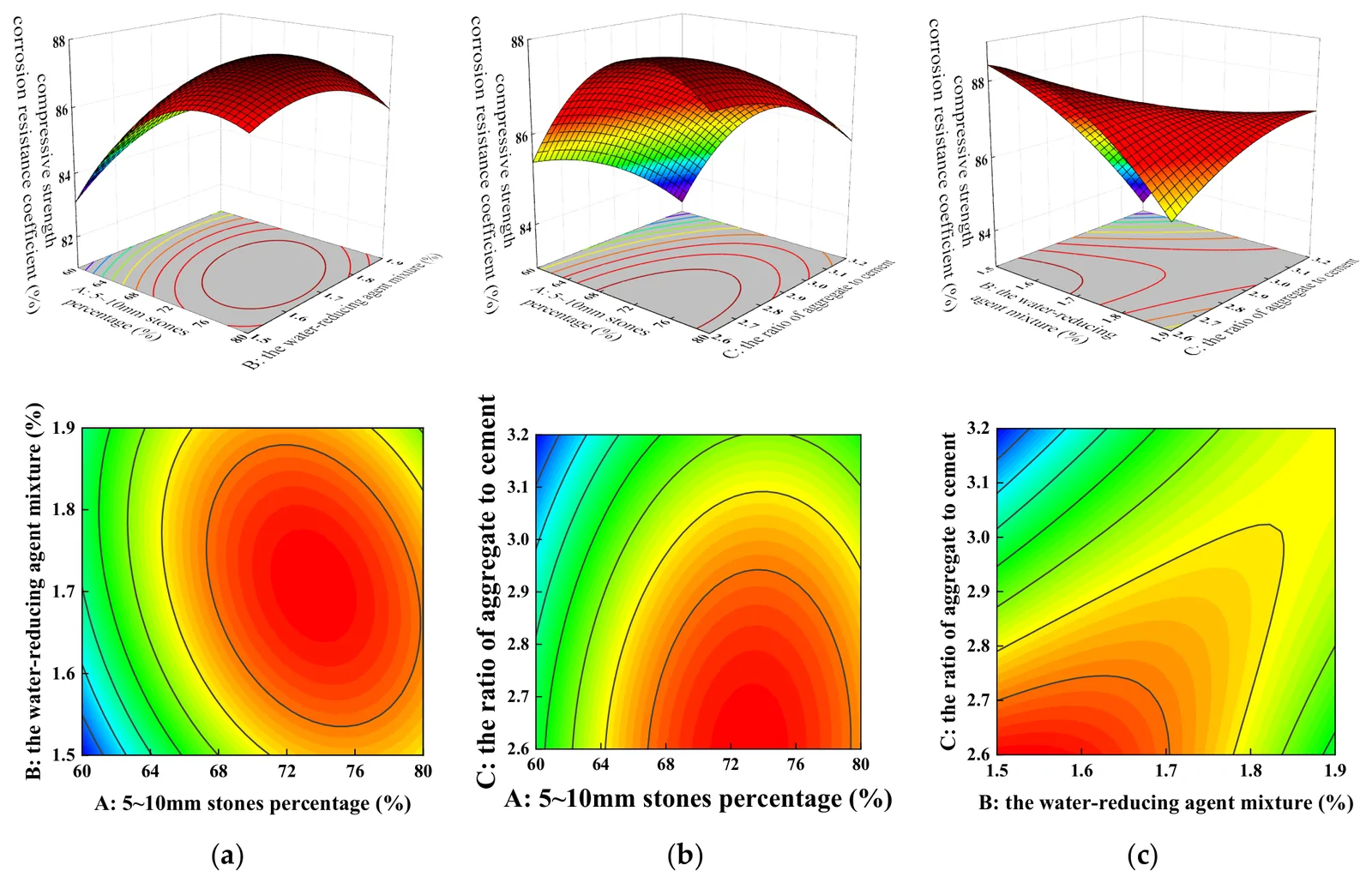
3D response surfaces and contour plots of compressive strength retention ratio under sulfate attack: (**a**) A–B interaction, (**b**) A–C interaction, and (**c**) B–C interaction.

Figure 4 shows the 3D response surface and contour plot of the compressive strength retention ratio under sulfate attack. In Figure 4a, the response surface exhibits an umbrella shape, and the compressive strength retention ratio under sulfate attack increases and then decreases with an increase in both the percentage of 5–10 mm stones and the water-reducing agent mixture. The larger curvature and denser contour lines along the coarse aggregate gradation axis indicate that its effect is greater than that of the water-reducing agent mixture.

Figure 4b shows that the compressive strength retention ratio under sulfate attack first increases and then decreases with an increase in both the percentage of 5–10 mm stones and the aggregate-to-cementitious material ratio. The contour plots reveal a significant interaction between the percentage of 5–10 mm stones and the aggregate-to-cementitious material ratio, with coarse aggregate gradation having a more pronounced effect.

Figure 4c illustrates that an increase in the water-reducing agent mixture increases and then decreases the compressive strength retention ratio under sulfate attack, whereas an increase in the aggregate-to-cementitious material ratio gradually decreases it. The larger curvature and denser contour lines for the aggregate-to-cementitious material ratio indicate that its influence is greater than that of the water-reducing agent mixture.

In summary, the influence on the compressive strength retention ratio under sulfate attack of flowable concrete is ranked as A > C > B, indicating that increasing both the proportion of 5–10 mm stones and the aggregate-to-cementitious material ratio can effectively improve this property. According to the ANOVA results in Table 6, the BC interaction term shows the lowest *p*-value (0.0004) among the three interaction terms for the compressive strength retention ratio under sulfate attack. The response surfaces in Figure 4 illustrate the modeled interaction trends.

(5) Analysis of relative mass loss ratio

**Figure 5:**
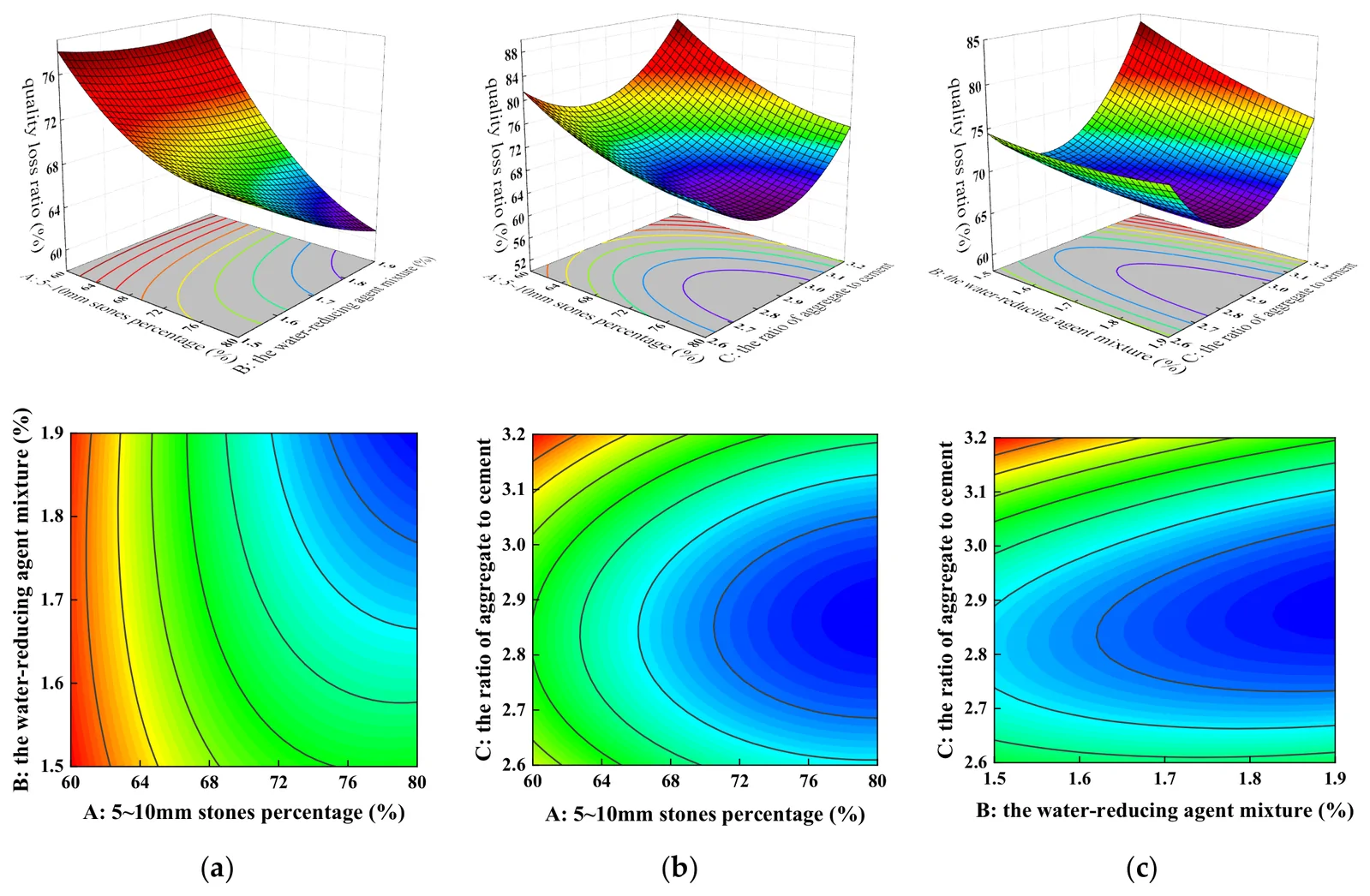
3D response surfaces and contour plots of relative mass loss ratio: (**a**) A–B interaction, (**b**) A–C interaction, and (**c**) B–C interaction.

Figure 5 shows the 3D response surface and contour plot of the relative mass loss ratio. In Figure 5a, the response surface displays a left-high to right-low inclined shape, with the relative mass loss ratio gradually decreasing as the percentage of 5–10 mm stones increases, while it gradually increases with an increase in the water-reducing agent mixture. According to the ANOVA results in Table 6, factor A and factor C both have statistically significant effects on the 90-day compressive strength retention ratio under sulfate attack, while factor B does not. The effect of coarse aggregate gradation is therefore ranked first, followed by the aggregate-to-cementitious material ratio.

Figure 5b demonstrates a significant interaction effect between the percentage of 5–10 mm stones and the water-reducing agent mixture on the relative mass loss ratio, with the ratio showing a trend of decreasing and then increasing as both variables increase. The contour lines on the side representing coarse aggregate gradation are denser than those on the side corresponding to the aggregate-to-cementitious material ratio, indicating that the former has a greater effect on the relative mass loss ratio.

In Figure 5c, an increase in the water-reducing agent mixture leads to a gradual decrease in the relative mass loss ratio, while an increase in the aggregate-to-cementitious material ratio causes an initial decrease followed by an increase in the relative mass loss ratio. The contour plot illustrates an interaction trend between the water-reducing agent mixture and the aggregate-to-cementitious material ratio. However, according to the ANOVA results in Table 6, the *p*-value for the BC interaction term is 0.0768 for the mass loss ratio, indicating that it is not statistically significant.

In summary, the influence on the relative mass loss ratio of flowable concrete is ranked as A > C > B, implying that increasing both the proportion of 5–10 mm stones and the aggregate-to-cementitious material ratio can effectively reduce the relative mass loss ratio. The ANOVA *p*-value verifies this conclusion. According to the ANOVA results in Table 6, the BC interaction term shows the lowest *p*-value among the three interaction terms for the mass loss ratio, though none of the interaction terms are statistically significant at the 0.05 level. The response surfaces in Figure 5 illustrate the modeled trends.

#### 3.1.3. Optimization of Mixing Ratios

The maximum values of slump flow, J-ring extensibility, 28-day compressive strength, and compressive strength retention ratio under sulfate attack, as well as the minimum value of the relative mass loss ratio, were optimized to obtain the optimum composite mix for workability, compressive strength, and sulfate corrosion resistance. The optimization was performed with the following constraints: slump flow, J-ring extensibility, 28-day compressive strength, and 90-day compressive strength retention ratio under sulfate attack were set to maximize, while the 90-day mass loss ratio was set to minimize. The overall desirability was 0.874. A verification test using the optimized mix gave measured values in good agreement with the model predictions, with relative errors ranging from 0.6% to 4.3%. The optimum mix proportions were as follows: the percentage of 5–10 mm stone was 71.149%, the water-reducing agent mixture was 1.695%, and the aggregate-to-cementitious material ratio was 2.749.

### 3.2. Microstructure Analysis

The groups with the highest and lowest compressive strength retention ratio under sulfate attack and relative mass loss ratio from the 90-day sulfate attack resistance test were selected. Group 6 had the highest compressive strength retention ratio under sulfate attack (88.3%), while Groups 2 and 12 had the lowest (83.1%); Group 2 exhibited the highest relative mass loss ratio (0.89) and Group 8 the lowest (0.62). Therefore, in this section, the immersion and water groups of Groups 2, 6, 8, and 12 were selected to perform the SEM and XRD tests.

#### 3.2.1. SEM Microscopic Morphology Analysis

**Figure 6:**
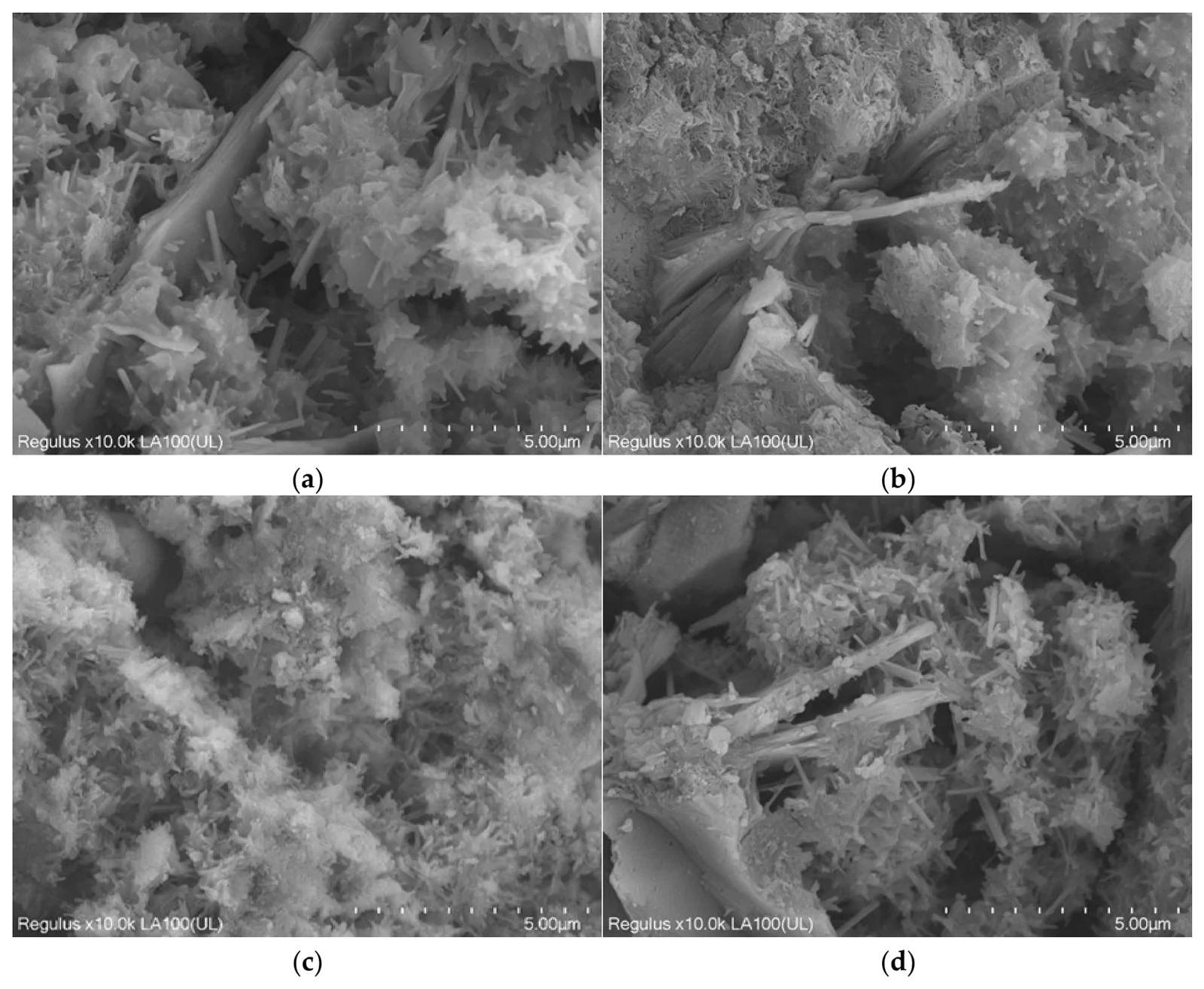
Microscopic morphology of the immersion group after 90 days. (**a**) Group 2, (**b**) Group 6, (**c**) Group 8, (**d**) Group 12.

**Figure 7:**
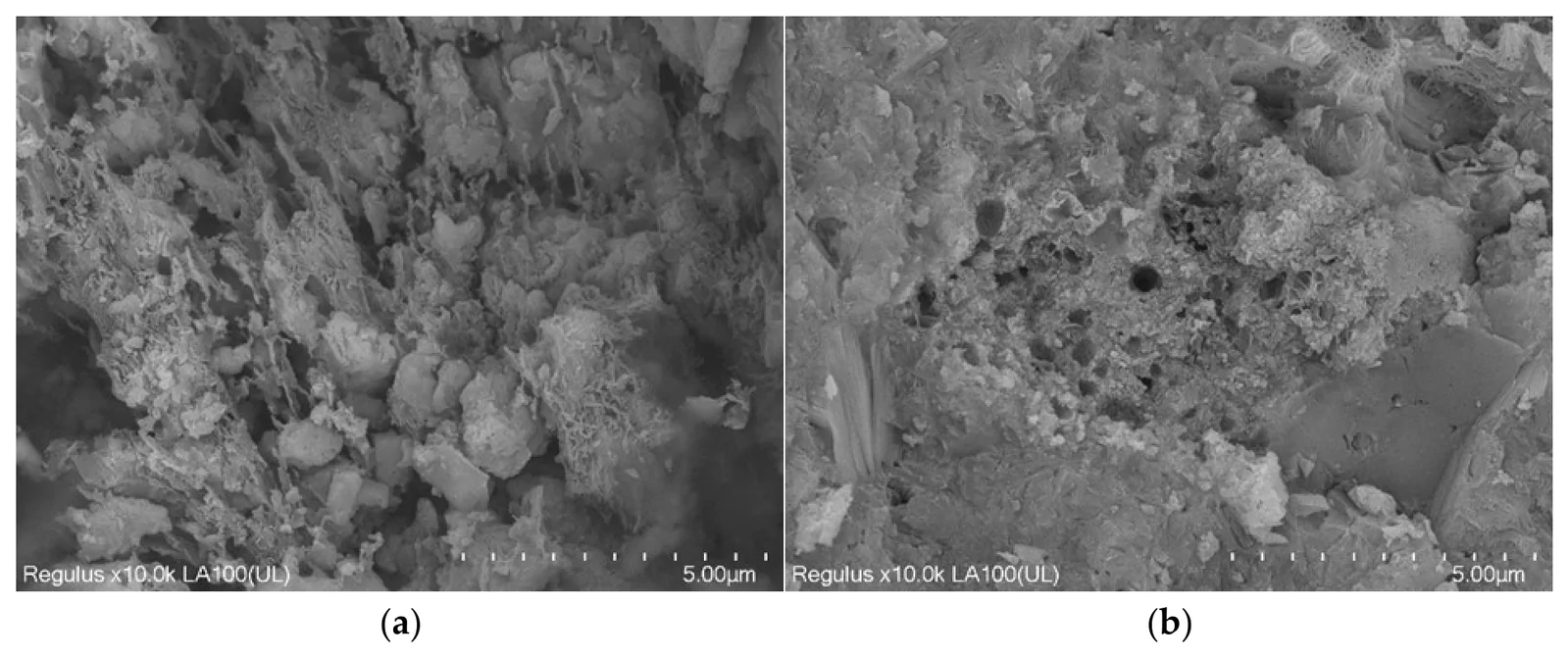

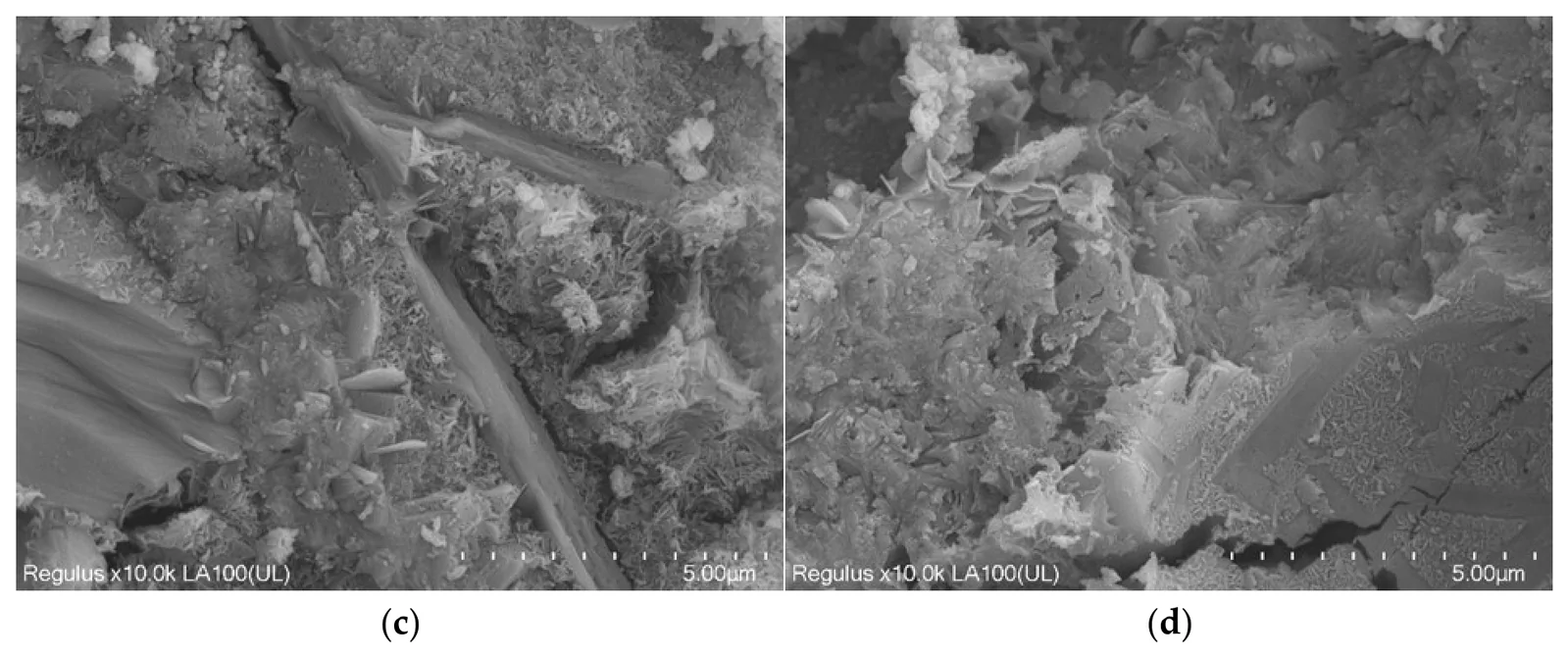
Microscopic morphology of the clear-water group. (**a**) Group 2, (**b**) Group 6, (**c**) Group 8, (**d**) Group 12.

The SEM images were used to characterize the microstructural morphology of the concrete specimens. As shown in Figure 6 and Figure 7, hexagonal flake-like crystals of portlandite, needle-like crystals of ettringite, and crystals of calcite intertwined with C-S-H gel were clearly observed in all the immersion groups. Hexagonal flake-like crystals with a parallel distribution were also observed. The C-S-H gel exhibited an irregular morphology. These microstructural features were more pronounced in Groups 2 and 12 than in Groups 6 and 8 and were more evident in the 90-day immersion group in Figure 6 than in the 90-day water group in Figure 7.

#### 3.2.2. XRD Pattern Analysis

**Figure 8:**
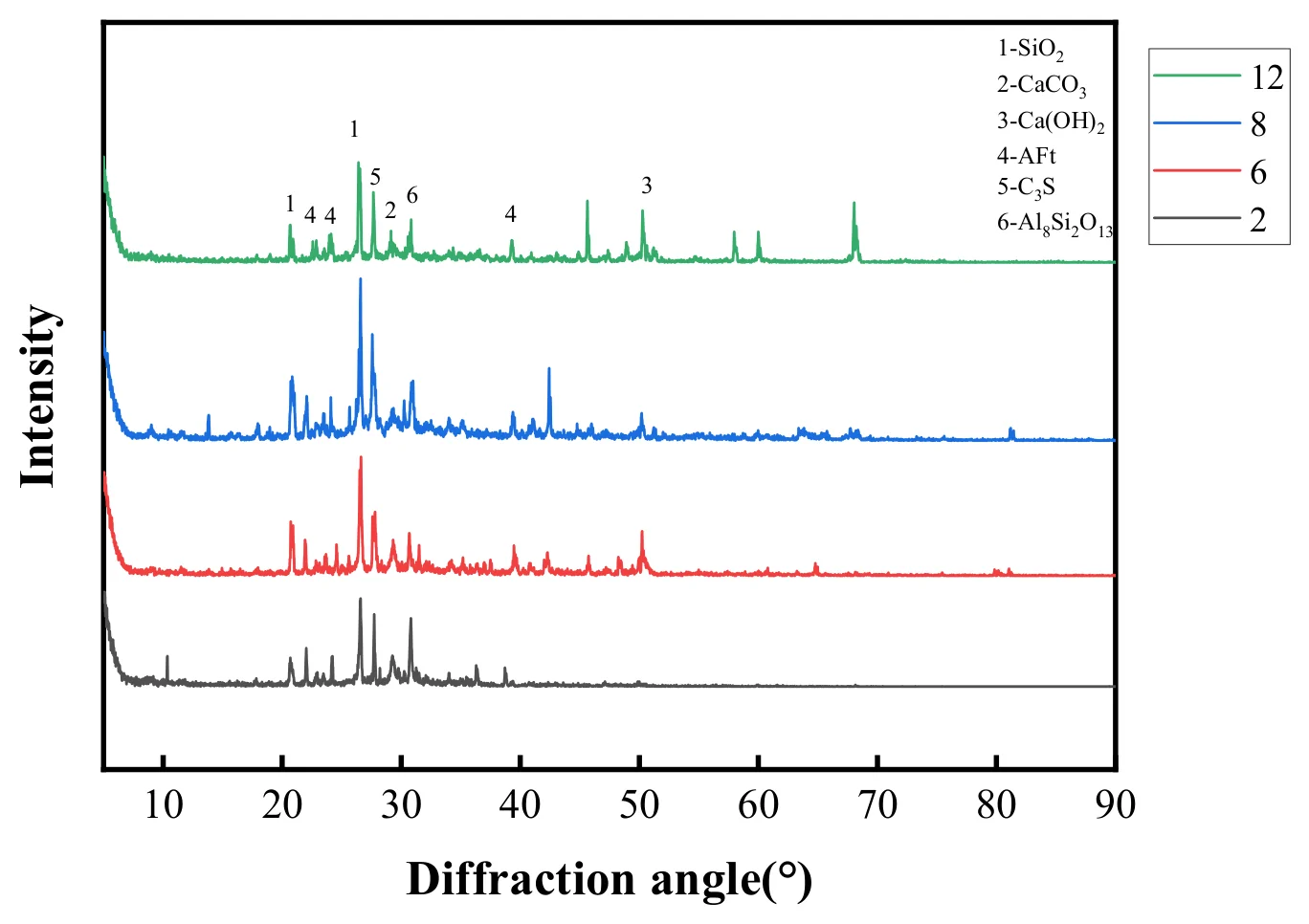
XRD pattern of the immersion group after 90 days.

**Figure 9:**
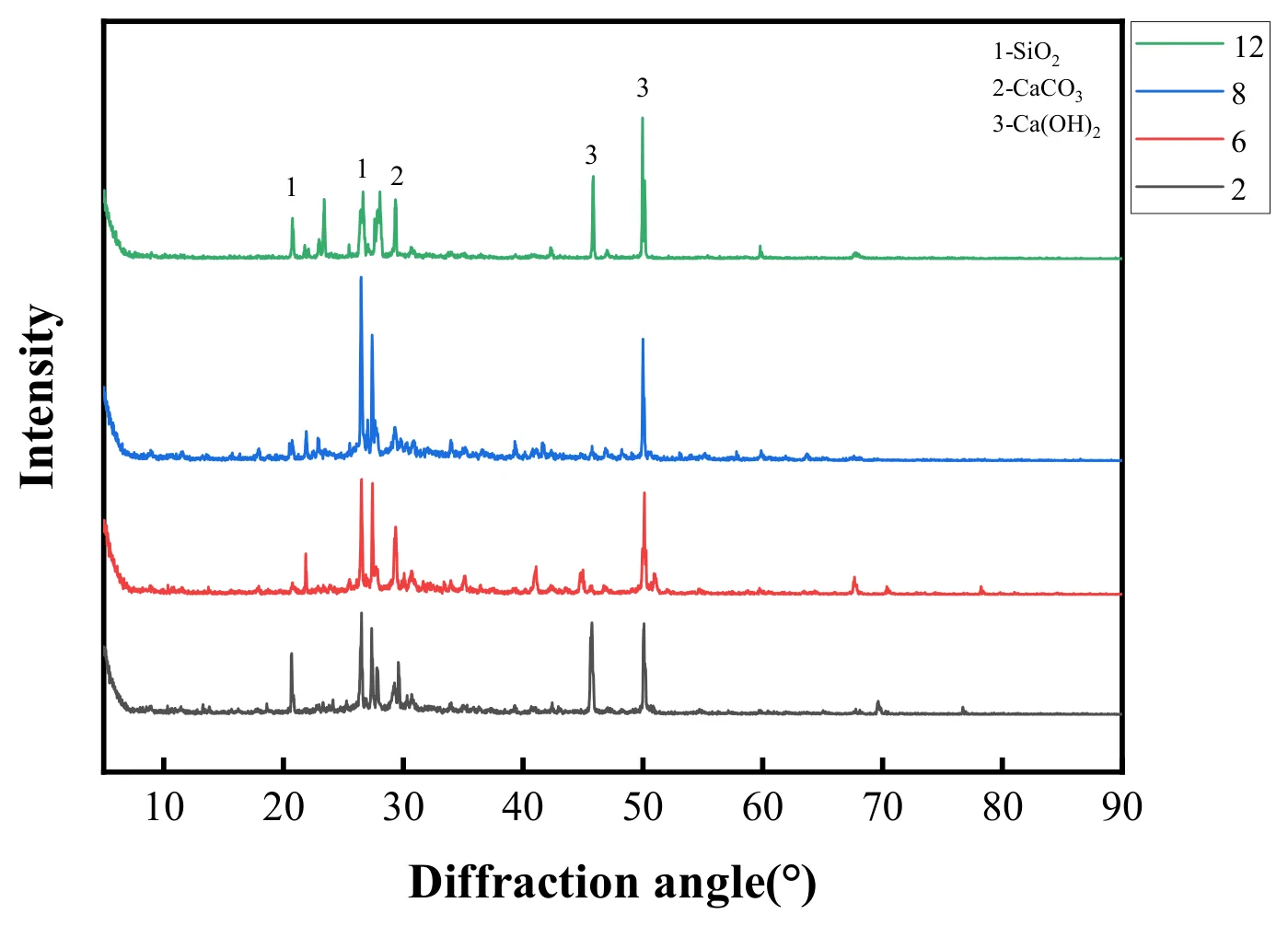
XRD map of the clear-water group.

Figure 8 and Figure 9 show the XRD patterns of concrete from the 90-day immersion and clear-water groups, respectively. As illustrated in Figure 8, the primary constituents of concrete in the 90-day immersion group were silica (SiO_2_), limestone (CaCO_3_), and calcium hydroxide (Ca(OH)_2_). As shown in Figure 9, the primary components of concrete in the clear-water group were silica (SiO_2_), limestone (CaCO_3_), and calcium hydroxide (Ca(OH)_2_). A comparison of the Ca(OH)_2_ diffraction peaks in the two figures shows that the intensity in the immersion group was lower than that in the clear-water group. This observation indicates that a portion of the Ca(OH)_2_ in the immersion group underwent chemical reactions with sulfate ions. Comparison of the XRD patterns in Figure 8 shows that the Ca(OH)_2_ diffraction peak intensity of Group 2 is lower than that of the other immersion groups, whereas Group 6 shows a relatively higher Ca(OH)_2_ peak intensity. This is consistent with the macroscopic results, where Group 2 exhibits a lower compressive strength retention ratio under sulfate attack and a higher mass loss ratio, while Group 6 shows a higher retention ratio.

### 3.3. Chemical Degradation Pathway Analysis

Sulfate attack on flowable concrete can involve a sequence of pore filling and subsequent expansion-induced deterioration. During the early stage, sulfate ions react with hydration products, particularly Ca(OH)_2_ and aluminate-bearing phases, leading to the formation of sulfate-related products such as gypsum and ettringite, as reported in previous studies [32]. These products may partially fill pores and microcracks, thereby increasing matrix compactness. This mechanism is consistent with the mass gain and increased compressive strength retention ratio observed during the early stage of sulfate exposure. However, the present XRD patterns do not provide unambiguous evidence for gypsum formation; therefore, the gypsum-related reaction is discussed here based on the established sulfate-attack mechanism rather than direct phase identification in the present XRD results.

As shown in Table 4, the compressive strength retention ratio under sulfate attacks exceeded 100% at 30 days for most groups, indicating that the formation of gypsum and ettringite at this early stage primarily filled pores and enhanced matrix compactness rather than causing damage. For example, Groups 6 and 14 reached 104.5% and 107.4% at 30 days, respectively. However, by 90 days, the coefficients declined to 83–88%, reflecting the transition from densification to deterioration. The continued formation of expansive products within the confined pores generated internal stresses, while the consumption of Ca(OH)_2_ weakened the cementitious matrix. These combined effects led to microcrack propagation and eventual surface spalling, as evidenced by the positive mass loss ratios observed from 70 to 80 days.

The mass loss ratio in Table 5 shows a non-monotonic trend over time. The negative values up to 60 days indicate a net mass gain due to the formation and accumulation of sulfate-related reaction products within the pores. As corrosion proceeds beyond 60 days, the ratio turns positive between 70 and 80 days, reflecting the onset of surface spalling and true mass loss caused by expansive cracking. This transition marks the shift from pore filling to deterioration.

At the middle and late stages from 60 to 90 days, continued formation and accumulation of sulfate-related reaction products within the confined pores can generate internal stresses, while consumption of Ca(OH)_2_ weakens the matrix. These effects initiate and propagate microcracks. Consequently, the retention ratio under sulfate attack decreases to 83–88% at 90 days as shown in Table 4, and the mass loss ratio turns from negative to positive between 70 and 80 days as shown in Table 5. The SEM and XRD results in Figure 6 and Figure 8 are consistent with this interpretation, with the more severely corroded groups exhibiting pronounced microcracking, abundant ettringite crystals, and lower Ca(OH)_2_ peak intensities.

## 4. Limitations and Future Perspectives

### 4.1. Contribution to the Existing Literature

Previous studies on sulfate resistance mainly focused on single factors or isolated durability indicators. This study fills this gap by systematically investigating the interactive effects of coarse aggregate gradation (A), water-reducer dosage (B), and aggregate-to-cement ratio (C) on both fresh properties and sulfate resistance using response surface methodology.

### 4.2. Limitations

First, sulfate corrosion tests were conducted under a fixed concentration (5% Na_2_SO_4_) and ambient temperature, whereas practical conditions often involve chloride coexistence, wetting–drying cycles, and temperature variations. Second, the 90-day corrosion period may not capture long-term (>1 year) performance evolution. Third, the economic and environmental aspects of the optimized mix were not assessed.

### 4.3. Future Perspectives

Future work should: (a) extend the response surface framework to multi-hazard scenarios (sulfate–chloride–freeze–thaw coupling); (b) validate the optimized mix with supplementary cementitious materials (e.g., slag, limestone powder) for low-carbon concrete; (c) incorporate life-cycle assessment (LCA) to balance performance, cost, and environmental impact; and (d) develop in situ monitoring indicators based on the mass loss turning point (70–80 d) identified in this study for early damage warning.

## 5. Conclusions

(1) The response surface method was used to optimize the mix design of flowable concrete. The established regression model accurately described the relationship between coarse aggregate gradation, water-reducing agent mixture, and aggregate-to-cementitious material ratio and the performance indices (slump flow, J-ring extensibility, 28-day compressive strength, compressive strength retention ratio under sulfate attack, and relative mass loss ratio). The model exhibited high credibility based on significance and model adequacy analyses.
(2) Response surface analysis showed that coarse aggregate gradation (A) had the most significant effect on the performance indices. An increase in the proportion of 5–10 mm stones improved slump flow and 28-day compressive strength, whereas an increase in the aggregate-to-cementitious material ratio (C) decreased 28-day compressive strength. The aggregate-to-cementitious material ratio (C) also played an important role in sulfate corrosion resistance, while the water-reducing agent mixture (B) had a relatively limited effect and, when excessive, could cause a reduction in strength.
(3) Through response surface model optimization, the optimal mix parameters for flowable concrete were determined as follows: 71.149% for 5–10 mm stones, 1.695% for the water-reducing agent mixture, and an aggregate-to-cementitious material ratio of 2.749. Verification test results confirmed the reliability of the optimization, with measured values in good agreement with the model predictions.
(4) Microanalysis showed that, at the initial stage of sulfate corrosion, the formation of ettringite and gypsum filled the pore space and enhanced compactness, which was macroscopically manifested as an increase in mass and compressive strength retention ratio under sulfate attack. However, the subsequent expansion of corrosion products triggered the expansion of microcracks, accelerating quality loss and strength deterioration. XRD and SEM tests verified the direct correlation between the corrosion products and concrete performance degradation.

## Figures and Tables

**Table 1:** Test factors and levels.

Code	Factor	Level		
		−1	0	1
A	Percentage of 5–10 mm stones (%)	60	70	80
B	Water-reducing agent mixture (%)	1.5	1.7	1.9
C	Aggregate-to-cementitious material ratio	2.6	2.9	3.2

**Table 2:** Specifications of flowable concrete Units: kg/m^3^.

Serial No.	Influencing Factors			Mix Proportions							
	Percentage of 5–10 mm Stones(A)	Water-Reducing Agent Mixture(B)	Aggregate-To-Cementitious Material Ratio(C)	Cement	Fly Ash	Microsilica Ash	Granule	5–10 mm Stone	10–20 mm Stone	Water	Water-Reducing Agent
1	70%	1.7%	2.9	402	115	57	766	629	270	195	10
2	60%	1.7%	3.2	377	108	54	793	559	372	183	9
3	70%	1.9%	2.6	430	123	61	735	604	259	209	12
4	80%	1.7%	2.6	430	123	61	735	691	173	209	10
5	70%	1.7%	2.9	402	115	57	766	629	270	195	10
6	70%	1.5%	2.6	430	123	61	735	604	259	209	9
7	60%	1.7%	2.6	430	123	61	735	518	345	209	10
8	80%	1.9%	2.9	402	115	57	766	719	180	195	11
9	70%	1.7%	2.9	402	115	57	766	629	270	195	10
10	60%	1.9%	2.9	402	115	57	766	540	360	195	11
11	70%	1.7%	2.9	402	115	57	766	629	270	195	10
12	60%	1.5%	2.9	402	115	57	766	540	360	195	9
13	70%	1.9%	3.2	377	108	54	793	652	279	183	10
14	80%	1.5%	2.9	402	115	57	766	719	180	195	9
15	70%	1.7%	2.9	402	115	57	766	629	270	195	10
16	80%	1.7%	3.2	377	108	54	793	745	186	183	9
17	70%	1.5%	3.2	377	108	54	793	652	279	183	8

**Table 3:** Workability and compressive strength.

Serial No.	Slump Flow (mm)	J-Ring Extensibility (mm)	28-Day Compressive Strength (MPa)
1	660	635	67.1
2	635	615	52.3
3	660	635	60.1
4	625	590	66.2
5	670	645	66.7
6	630	610	65.7
7	655	630	62.4
8	605	570	63.7
9	670	635	63.8
10	630	605	57.2
11	665	645	64.9
12	620	600	53.5
13	640	605	63.3
14	595	575	63.4
15	675	655	63.5
16	620	585	63.2
17	635	610	52.3

**Table 4:** Compressive strength retention ratio under sulfate attack.

Serial No.	30 Days			60 Days			90 Days		
	Compressive Strength (MPa)		Corrosion Resistance (%)	Compressive Strength (MPa)		Corrosion Resistance (%)	Compressive Strength (MPa)		Corrosion Resistance (%)
	Immersion Group	Water Group		Immersion Group	Water Group		Immersion Group	Water Group	
1	65.7	64.2	102.3	65.1	66.7	97.6	60.5	69.6	86.9
2	54.4	52.3	103.1	49.4	54.2	94.6	44.6	55.7	83.1
3	55.4	60.9	102.7	51.4	62.3	96.4	54.3	63.5	85.5
4	56.7	65.4	104.2	58.3	66.3	95.1	60.5	69.2	87.4
5	64.9	67.3	102.5	59.5	67.1	94.1	59	68.2	86.5
6	65.3	65.5	104.5	62.9	66.8	95.6	59.4	67.3	88.3
7	63.3	63.5	102.9	65.2	65.4	95.3	54.3	67.6	85.5
8	65.2	64.8	103.8	60.8	65.1	93.4	56.7	66.1	85.8
9	65.9	63.5	107.2	59.1	64.7	94.3	57.6	67.3	88.2
10	66.7	58.3	102.1	65.6	59.3	94.7	50.7	59.8	84.8
11	62.8	65.9	101.5	58.5	66.1	92.9	57.7	67.9	87.6
12	62.4	54.7	101.1	61.6	55.8	93.6	45.8	55.1	83.1
13	70.3	64.6	102.5	63.3	65.2	94.2	57.1	66.5	87.2
14	68.5	63.8	107.4	64.6	66.8	96.7	57.4	66.4	86.4
15	54.4	62.1	106.5	52.7	65.2	95.5	49.1	66.2	87.4
16	62.9	64.3	104.3	62.2	65.7	94.7	55.2	67.5	85.6
17	67.9	52.6	102	58.5	52.7	94.8	46.6	55.9	83.4

**Table 5:** Relative mass loss ratios for the 90-day group.

Serial No.	10 Days	20 Days	30 Days	40 Days	50 Days	60 Days	70 Days	80 Days	90 Days
1	−0.39	−0.78	−1.13	−1.09	−0.74	−0.34	0.01	0.36	0.71
2	−0.29	−0.46	−0.81	−1.16	−0.73	−0.33	0.10	0.49	0.89
3	−0.12	−0.45	−0.76	−1.07	−0.98	−0.50	−0.10	0.29	0.73
4	−0.22	−0.46	−0.73	−0.99	−0.90	−0.51	−0.03	0.32	0.71
5	−0.20	−0.48	−0.82	−1.16	−0.78	−0.35	−0.09	0.34	0.72
6	−0.26	−0.55	−0.73	−1.09	−1.27	−0.73	−0.24	0.25	0.75
7	−0.21	−0.63	−0.90	−1.21	−0.77	−0.32	0.07	0.43	0.82
8	−0.19	−0.45	−0.70	−1.09	−1.09	−0.66	−0.28	0.19	0.62
9	−0.22	−0.49	−0.93	−1.15	−0.97	−0.62	−0.13	0.31	0.66
10	−0.29	−0.44	−0.84	−0.92	−0.97	−0.57	−0.09	0.34	0.78
11	−0.29	−0.56	−0.78	−1.00	−0.86	−0.56	−0.12	0.23	0.67
12	−0.28	−0.64	−0.94	−1.23	−0.85	−0.47	−0.09	0.38	0.77
13	−0.29	−0.59	−0.89	−1.24	−0.80	−0.46	−0.03	0.36	0.75
14	−0.30	−0.52	−0.86	−1.21	−0.91	−0.52	−0.09	0.30	0.69
15	−0.13	−0.33	−0.64	−0.90	−1.12	−0.59	−0.20	0.24	0.64
16	−0.19	−0.49	−0.66	−0.79	−1.17	−0.66	−0.15	0.32	0.74
17	−0.30	−0.59	−0.85	−1.19	−0.85	−0.50	−0.02	0.46	0.85

**Table 6:** Analysis of response surface modeling significance.

Form	Slump Flow	J-Ring Extensibility	28-Day Compressive Strength	Compressive Strength Retention Ratio Under Sulfate Attack	Relative Mass Loss Ratio
Model	0.0001	0.0002	0.0002	0.0005	0.0063
A-Coarse aggregate gradation	0.0003	0.0003	0.0001	0.0005	0.0014
B-Water-reducing agent mixture	0.0137	0.3538	0.0415	0.1904	0.114
C-aggregate-to-cementitious material ratio of	0.0347	0.0421	0.0005	0.0014	0.0187
AB	0.6581	0.5053	0.2432	0.0596	0.6468
AC	0.2083	0.5053	0.0324	0.5764	0.5437
BC	0.0542	0.0732	0.0004	0.0004	0.0768
A^2^	0.0001	0.0001	0.0059	0.0005	0.1457
B^2^	0.0001	0.0003	0.0017	0.0152	0.6384
C^2^	0.2414	0.1741	0.0399	0.1343	0.0017
Loss of proposed item	0.5682	0.788	0.8884	0.9561	0.8226
R^2^	0.9773	0.9660	0.9678	0.9592	0.9386
R_a_^2^	0.9480	0.9223	0.9265	0.9067	0.8596
R_p_^2^	0.8443	0.8432	0.8879	0.8953	0.8093

## Data Availability

The raw data supporting the conclusions of this article will be made available by the authors on request.
